# Transforming Gas Separations with Scalable Carbon Molecular Sieve Membranes Enabled by Chemically Tuned Hollow‐Fiber Precursors

**DOI:** 10.1002/advs.78223

**Published:** 2026-10-09

**Authors:** Yuhe Cao, Zhongyun Liu, Zahra Shomali, Jinyoung Kim, Steven Schlosser, Ryan P. Lively, William J. Koros

**Affiliations:** ^1^ School of Chemical & Biomolecular Engineering Georgia Institute of Technology Atlanta Georgia USA; ^2^ Department of Civil Environmental and Construction Engineering The University of Texas at El Paso El Paso Texas USA; ^3^ Key Lab of Functional Polymers for Sustainability of Jiangsu School of Energy and Environment Southeast University Nanjing Jiangsu People's Republic of China

**Keywords:** Carbon molecular sieve, helium separation, hollow fiber membrane, hydrogen separation, ultraselective

## Abstract

Blending hydrogen into natural gas pipelines requires efficient separation, while helium recovery from natural gas via cryogenic distillation is energy‐ and capital‐intensive, driving demand for better membranes. This study develops ultraselective carbon molecular sieve (CMS) hollow fiber membranes from chemically modified Matrimid (pyrolyzed at 900°C). Two types of modifications are reported for both applications: treatment with vinyltrimethoxysilane (VTMS) or with diethyltoluenediamine/trimesoyl chloride (DETDA/TMC) and VTMS. The chemical modifications create highly economical new precursor hollow fibers that cannot be formed via conventional solvent dissolution and spinning. This establishes a broadly tunable network‐polymer platform for polyimide‐based precursor hollow fiber membranes, extendable to various diamines, which represents a transformative materials platform for scalable CMS membranes. Superior gas separation performance has been achieved, which is envisioned to be extendable across many gas pair cases. For 10% H_2_/90% CH_4_ feeds, the membrane achieves 99.75% H_2_ purity and 15.6 GPU H_2_ permeance, enabling dual‐use hybrid pipelines. For He recovery, CMS membranes stabilized by VTMS avoid pyrolysis‐induced collapse, and VTMS treatment delivers 18.6 GPU He permeance with high selectivity. Alternatively, the DETDA/TMC+VTMS treatment thins the dense layer (to ∼1 µm), boosting permeance under mixed feeds (8.7 vs. 6.0 GPU) while retaining ∼99% He purity.

## Graphical abstract

We established a broadly tunable network‐polymer platform for polyimide‐based precursor membranes, extendable to various diamines. Ultrahigh selectivity with demonstrated aging resistance, and scalability via hollow fiber CMS engineering collectively establishes broad and lasting impact for the membrane science and engineering communities.

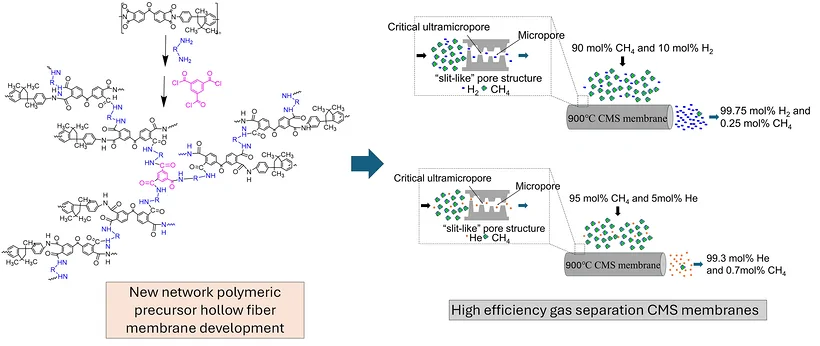

## 1 Introduction

Hydrogen produced by diverse methods [1, 2, 3, 4, 5, 6] is a low‐carbon energy carrier and chemical feedstock. While offering a path to reduce carbon emissions from the chemicals and energy sectors, large‐scale hydrogen transport currently requires a costly dedicated distribution infrastructure [1, 2]. Blending hydrogen into existing natural gas pipelines offers a potentially safe and economical alternative for hydrogen distribution [1, 2]. In 2019, the International Energy Agency (IEA) noted 37 demonstration projects investigating integration of hydrogen into natural gas networks [3]. One key challenge with blending hydrogen into natural gas pipelines is hydrogen embrittlement of the metals in the pipelines. To mitigate this embrittlement effect, blending ratios must be very low; i.e., hydrogen concentrations of 10 mol% or less in natural gas are likely to be required. Economical recovery of high‐purity hydrogen after dilution into the blended gas pipeline requires efficient point‐of‐use purification technologies, which currently do not exist. Ultrahigh permselectivity carbon molecular sieve (CMS) hollow fiber membranes can enable this blended gas distribution vision. Such membranes have ultrahigh gas permselectivities [4] and appealing productivities suitable to deliver hydrogen with high purities for various stringent application requirements. As is shown in Figure 1a, hydrogen extraction modules (HEMs) enable the natural gas pipeline network to serve as a hybrid carrier, allowing high‑purity H_2_ to be extracted at points of use. In this vision, the pipeline itself functions not merely as a transport conduit, but as an integral component of a distributed separation system. In fact, CMS membranes have already been investigated for hydrogen separation from hydrogen–natural gas blends. A dense flat‐sheet cellulose‐derived CMS membrane exhibited an H_2_/CH_4_ selectivity of 3,498 [7]. Composite‐supported alumina–CMS membranes achieved an H_2_ permeability of 750 Barrer and an H_2_/CH_4_ selectivity of 1,540 [8]. In addition, a cellulose‐derived hollow‐fiber CMS membrane exhibited a pure‐gas H_2_/CH_4_ selectivity of 5506 at 130°C [9]. Although these studies demonstrate the potential of CMS membranes for H_2_/CH_4_ separation, scalable and cost‐effective fabrication remains an important challenge. Higher selectivity is critical for on‐site H_2_ recovery from natural gas networks. Here, we report a straightforward and scalable approach for producing CMS membranes in a hollow‐fiber configuration, which is particularly advantageous for membrane scale‐up because of its high packing density. The precursor hollow fibers are fabricated using cost‐effective Matrimid and a relatively simple DETDA/TMC treatment, eliminating the need for expensive ionic‐liquid solvents or fluorinated polyimide precursors. The resulting membranes exhibit substantially higher H_2_/CH_4_ selectivity than previously reported hollow fiber CMS membranes. This combination of scalable fabrication, economical materials, straightforward chemistry, and strong separation performance highlights the potential of this membrane platform for commercial applications.

**FIGURE 1:**
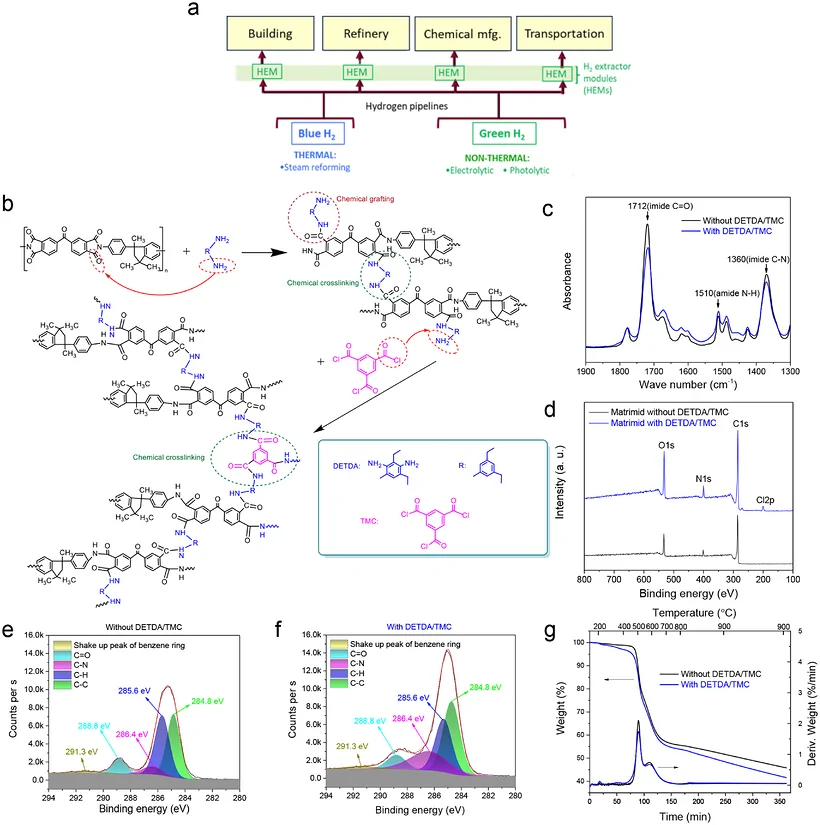
Preparation and characterization of Matrimid precursor hollow fiber membranes with and without DETDA/TMC modification. (a) HEM‐enabled H_2_ delivery natural gas pipeline network, (b) Expected modification mechanism of Matrimid with DETDA/TMC. Characterization of Matrimid precursor hollow fiber membranes with and without DETDA/TMC modification: (c) ATR‐FTIR spectra and (d) XPS survey spectra. High‐resolution C1s XPS spectra of Matrimid precursor fiber membranes: (e) without, and (f) with DETDA/TMC. (g) TGA of Matrimid precursor fiber membranes with and without DETDA/TMC.

Beyond hydrogen, another separation challenge, “selective recovery of a high‐value light gas from a predominantly methane matrix” arises in the context of helium (He). Global He demand has been increasing rapidly, by approximately 2 million m^3^ per year, driven by growth across diverse industrial, scientific, and medical applications [5, 6]. Commercial He relies almost exclusively on helium‐bearing natural gas, which can contain up to 8% helium [10, 11]. He recovery has been dominated by cryogenic distillation, which is both energy‐ and capital‐intensive processes [5]. Membrane‐based separation for He recovery has been investigated since the 1960s, with cellulose acetate among the earliest materials evaluated [12]. Because most reservoirs contain low He concentrations (0.21–4.05 mol%), membranes with very high He selectivity are required to achieve economically viable He purification. High He selectivity reduces compressor power and overall energy demand during the membrane operation [13] by minimizing the need for additional compression stages required to reach the target He purity. Conventional polymeric membranes suffer from an intrinsic selectivity‐permeability trade‐off, resulting in low He separation efficiency [6]. Zeolite membranes have high permeance with moderate He/CH_4_ selectivity of 83 [6]; however, CMS membranes have demonstrated ultra‐selective He/CH_4_ separation, achieving a pure‐gas selectivity of 16 700 [4]. This fact notwithstanding, the as‐prepared membranes exhibited low He permeance, attributed to structural collapse during pyrolysis.

A composite CMS membrane previously achieved He/CH_4_ selectivity of 137.07 with 1248.5 GPU He permeance [14]. However, higher selectivity is essential for producing high‐purity He from natural gas due to the low He concentrations in the feed stream. Our recent work demonstrated that a hyperaged CMS membrane prepared at a moderate pyrolysis temperature achieved He/CH_4_ selectivity of 1338 with 26 GPU He [15]. This study reports that DETDA/TMC‐treated CMS membranes achieve stabilized He/CH_4_ selectivity of 2001 with 7.6 GPU after 24 days of aggressive aging. Using 5/95 mol% He/CH_4_ feed mixtures, the CMS membranes reported herein achieved 99% He purity.

While conventional CMS fabrication relies on thermally labile supports that collapse during carbonization, our work presents a paradigm shift: direct chemical tuning of preformed hollow fibers into stabilized network precursors. By sequentially cross linking Matrimid with DETDA/TMC and stabilizing with VTMS sol–gel chemistry, we resolve the collapse‐stability trade‐off while preserving asymmetric morphology. This approach circumvents intractable challenges in spinning highly cross‐linked polymers directly. For decades, this limitation has severely constrained the pool of polymer precursors viable for CMS membrane development. It unlocks untapped potential in commercially available polyimides, establishing a versatile platform for network‐derived hollow fiber membrane precursors designed with tunable pyrolytical behavior and enhanced selectivity. In this study, a novel polymeric hollow fiber precursor membrane was fabricated via in situ DETDA/TMC cross‐linking of the entire as‐spun hollow fiber matrix, including both the selective separation layer and the porous support layer. We demonstrate that targeted chemical modification of CMS precursor hollow fibers using vinyltrimethoxysilane (VTMS) effectively mitigates this structural collapse. The resulting modified‐Matrimid derived CMS membranes exhibit significantly enhanced permeance while retaining, and in some cases improving, ultrahigh H_2_/CH_4_ and He/CH_4_ separation performance. By addressing the long‐standing permeance limitation of ultra‐selective CMS membranes, this work positions a single membrane platform as a versatile solution for both hydrogen extraction from blended natural gas pipelines and helium recovery from low‐concentration natural gas streams. The convergence of these two applications highlights a broader opportunity: the use of advanced molecular sieve membranes to give value, not by changing what flows through it, but by redefining what can be selectively removed. This simple but powerful new paradigm should be applicable across many gas pair types with high payoff impact.

## 2 Results and Discussion

### 2.1 H_2_/CH_4_ Separation

In this article, we report an economical thin selective layer Matrimid precursor hollow fiber, modified with controlled treatment using diethyltoluenediamine (DETDA) and trimesoyl chloride (TMC), followed by VTMS sol–gel stabilization to minimize membrane matrix collapse during pyrolysis at 900°C. The resultant CMS delivers hydrogen purity of 99.8 mol% with a permeance of 15.6 GPU (Gas Permeation Units, where 1 GPU = 10^−6^ cm^3^(STP) cm^−2^ s^−1^ cmHg^−1^) for a 10% H_2_/90% CH_4_ (mol/mol) feed mixture by strong exclusion of bulky CH_4_ molecules [4]. Mixed gas results are also reported with only VTMS treatment to illustrate the ultimate advantage of the dual DETDA/TMC and VTMS treatments.

Figure 1b illustrates the above‐mentioned chemical modification of Matrimid polyimide using DETDA/TMC, via low‐concentration DETDA in hexane. Besides possible cross‐linking, chemical grafting may occur when amine groups react with an imide during immersion of the precursor fiber membranes in a 2% diamine/hexane solution. Simple subsequent hexane rinsing removes residual DETDA, and a 2% TMC/hexane solution contact achieves the desired modifications. This scalable approach creates a systematically controllable packing‐disrupting network polymer precursor hollow fiber without destroying the membrane asymmetry. Figure 1c presents the Fourier transform infrared (FTIR) spectra of Matrimid membranes before and after modification with DETDA/TMC. The characteristic peaks at 1712 and 1360 cm^−^^1^ correspond to the symmetric carbonyl (C═O) stretching and C–N stretching vibrations of the imide groups in Matrimid [16]. Additionally, the peak at 1510 cm^−^^1^ is assigned to the N–H bending vibration of the amide groups [17]. To evaluate the extent of the chemical modification, changes in the amide peak at 1510 cm^−^^1^ and the imide peak at 1360 cm^−^^1^ were examined. The intensity ratio of these peaks (I_amide,1510_/I_imide,1360_) increased from 0.52 to 0.71 after DETDA/TMC modification, indicating the introduction of amide groups into the membrane.

Figure 1d shows the wide‐scan x‐ray photoelectron spectroscopy (XPS) spectra of fiber membranes with and without DETDA/TMC modification. Trace chlorine in the modified membrane arises from the HCl byproduct generated during the reaction between amine and acyl chloride [18, 19], which formed chloride salts via reaction with amine groups [20]. This trace residual chlorine does not present a problem in our application. The elemental composition of each membrane (as shown in Table S1) reveals a significant increase in the concentrations of nitrogen (N) and oxygen (O) following modification with DETDA/TMC. Specifically, the nitrogen concentration increased from 5.9 to 8.0 at. %, while the oxygen concentration rose from 14.9 to 18.5 at. %. Correspondingly, the O/C and N/C ratios also exhibited noticeable increases, from 0.19 and 0.07 to 0.26 and 0.11, respectively. These changes reflect incorporation of nitrogen from DETDA and oxygen from TMC during the modification process.

The C_1s_ core‐level spectra of the membranes (Figure 1e,f) were deconvoluted into five peaks, as shown in Table S2, which correspond to five distinct chemical bonds [21]. Following DETDA/TMC modification, the relative content of C‐N bonds increased significantly from 14.2% to 26.0%. Additionally, the relative content of C═O bonds also rose from 9.3% to 11.7%. These changes further substantiate the incorporation of ─NH_2_ and ─C═O functional groups into the membranes via the DETDA/TMC modification process, thereby creating a different precursor than the original Matrimid, but still with desirable membrane asymmetry.

The thermal stability and decomposition behavior of the precursor membranes were investigated using TGA‐FTIR (Figure 1g). Both precursor membranes exhibited similar mass loss trends; however, the DETDA/TMC‐modified precursor membrane displayed a 4.3% greater mass loss. Moreover, besides the typical CH_4_, CO_2_, and CO [22] gaseous decomposition products, the DETDA/TMC‐modified precursor also released amide‐related compounds (1750 cm^−^^1^) [23]. This suggests that DETDA/TMC modification leads to the evolution of additional volatile fragments, potentially promoting formation of a more porous carbon structure.

FTIR, XPS, and TGA‐FTIR analyses confirm DETDA/TMC incorporation through increased amide, nitrogen, oxygen, carbon–nitrogen, and carbonyl signatures. However, overlapping chemical features prevent absolute loading quantification. XPS verifies near‐surface modification, while bulk analyses suggest deeper incorporation, although the available data cannot establish conclusively uniform distribution throughout the dense selective layer.

The final anti‐collapse VTMS treatment further stabilizes properties even for a 24‐day aggressive vacuum aging case. The as‐spun morphology of the precursor fiber membranes, presented in Figure 2a–c, has a separation layer with an average thickness of ∼150 nm supported by a porous sublayer. In our prior case without the VTMS treatment, a similar porous support layer totally collapsed during pyrolysis [24], as shown in Figure S1, so the VTMS treatment is valuable in all cases.

**FIGURE 2:**
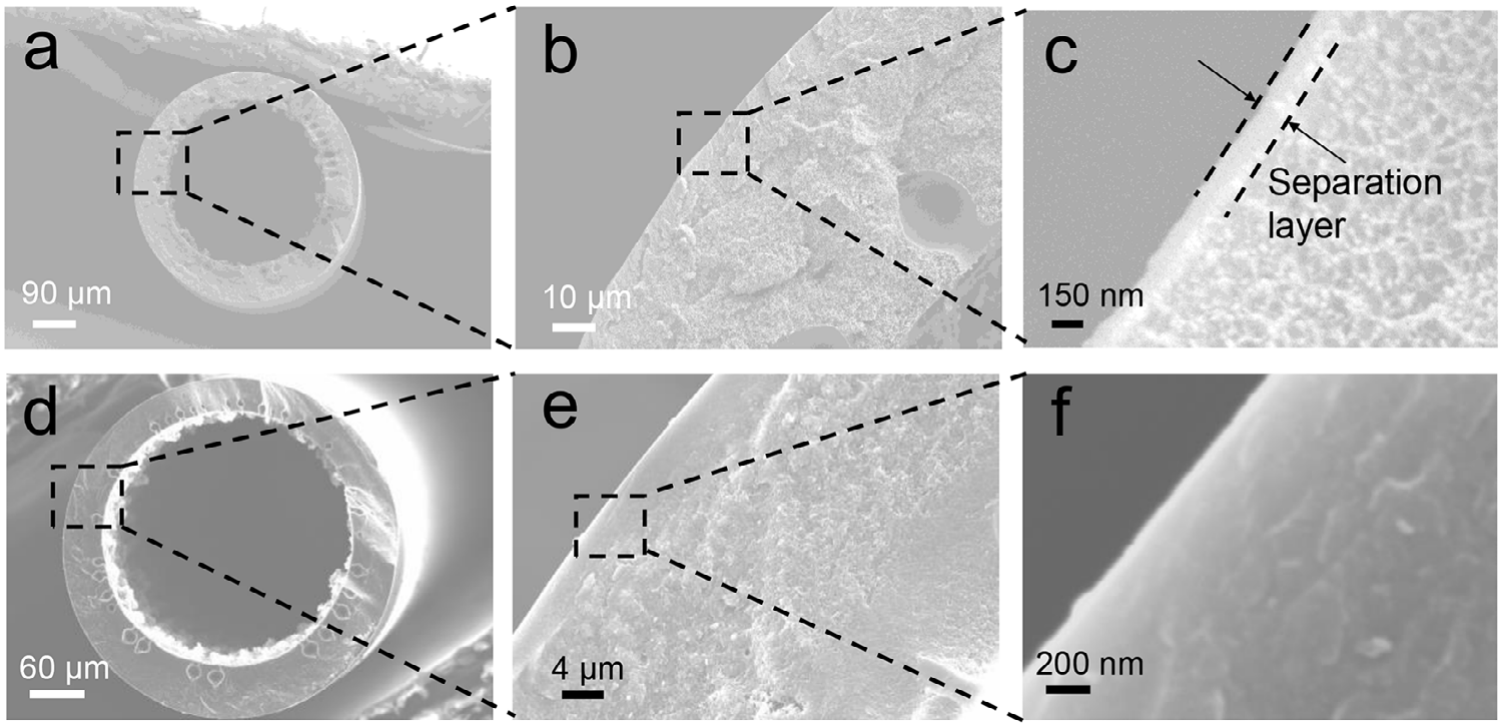
Scanning electron microscopy characterization of samples treated with DETDA/TMC followed by VTMS treatment. Matrimid Precursor: (a) cross‐section, (b) fiber wall, (c) top separation layer. Also, final 900°C pyrolyzed CMS: (d) cross‐section, (e) fiber wall, (f) top separation layer.

The carbon membrane fibers with dual DETDA/TMC and VTMS treatments retain a porous structure of the support layers (Figure 2e,f), enabling formation of a hierarchical architecture with both selective and porous domains for the CMS membrane prepared at 900°C, unlike the case without VTMS treatment in Figure S1.

Of course, the CMS membrane separation layer obtained with DETDA/TMC modification (Figure 2f) originates from the ∼150 nm top selective layer, along with a portion of the underlying support layer. As illustrated in Figure S2, as opposed to Figure S1 without the dual DETDA/TMC and VTMS treatments, the modified membrane retains a more open substrate pore structure beneath the dense separation layer. The VTMS treatment alone is highly effective in mitigating substrate collapse during high‑temperature pyrolysis, as evidenced in Figure S3. Without VTMS treatment, the completely densified fiber wall (shown in Figure S1) gives lower pure gas permeance and somewhat higher selectivity [4]. Here, VTMS‐treated CMS without DETDA/TMC gave a 5.5 times higher H_2_ permeance (43.8 GPU) shown in Table S3, than the corresponding 8 GPU permeance in the previous study with a similar 50 µm precursor thickness fiber wall (shown in Table S5) for the totally collapsed CMS with a 33 µm thick wall [4]. The morphology in molecular sieving selective walls [25] may experience very subtle packing disruptions occurring with VTMS treatments, causing somewhat reduced selectivity relative to the case without VTMS.

For the dual DETDA/TMC and VTMS treatments, the resultant dense CMS separation layer is thinner (∼1 µm) with a more pronounced porous substrate structure apparent in Figure S2b. Besides the anti‐collapse feature from VTMS, DETDA/TMC treatment also contributed to suppressing collapse of the transition layer during carbonization, leading to a thinner skin layer compared with CMS treated with VTMS alone. Moreover, subtle packing differences reflecting performance may exist compared to the case without DETDA/TMC and can be probed by H_2_/CH_4_ selectivity and H_2_ permeance.

Pure gas permeances and selectivity for the H_2_/CH_4_ gas pair are illustrated in Figure 3a and Table S3. The separation properties with only VTMS showed H_2_/CH_4_ selectivity of 14 592.8 and 43.8 GPU H_2_ permeance, compared to H_2_/CH_4_ selectivity of 5891.7 and 41.9 GPU H_2_ permeance after the dual DETDA/TMC and VTMS treatment. DETDA/TMC treatment introduces a higher density of cross‐linked sites into the precursor polymer chains. We hypothesize that upon pyrolysis, these cross‐linked domains promote stress‐induced increased chain scission, giving rise to shorter carbon strands relative to those formed from VTMS‐only‐treated Matrimid. We postulate that such shortened strands assemble into smaller carbon plates, which stack more densely to form a tighter and less permeable separation layer. Consistent with this structural evolution, slightly reduced pure gas permeances for H_2_, CO_2_, O_2_, and N_2_ are observed (Table S8). In contrast, the pure gas permeance of He remains essentially unchanged, likely reflecting its spherical molecular geometry and the minimal steric hindrance encountered during transport. The presence of imperfections in the separation layer caused by the above‐mentioned chain scission, carbon plate formation, and stacking facilitates enhanced CH_4_ permeance. This trend further shows the influence of DETDA/TMC with the VTMS; however, the CMS membranes with and without DETDA/TMC treatment both showed excellent pure gas selectivity and permeances in Figure 3a and Table S3 that are beyond the state‐of‐the‐art 2015 H_2_/CH_4_ pure gas upper bound [26]. Cellulose‐derived CMS membrane showed H_2_/CH_4_ pure gas selectivity of 5506 at 130°C [12], and based on reported temperature trends, its selectivity at 35°C would be significantly lower. By contrast, our membrane achieves H_2_/CH_4_ pure gas selectivity of 14 592.8 at 35°C, already far exceeding that benchmark under more practical operating conditions. Meanwhile, investigations of mixed gases (H_2_/CH_4_ and He/CH_4_) were not reported for the cellulose‐derived CMS membranes.

**FIGURE 3:**
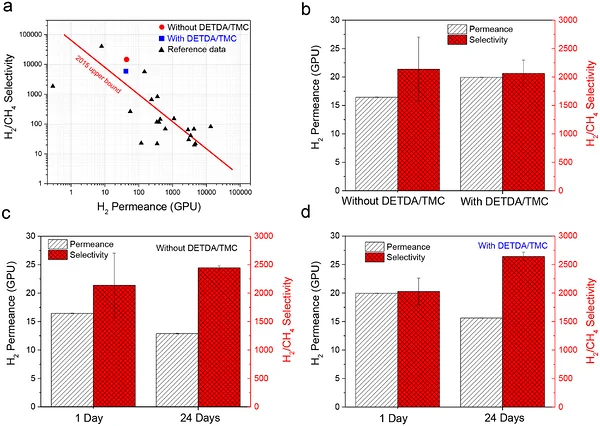
Gas separation performance of the prepared CMS hollow fiber membranes. (a) Pure gas separation performance comparison: without DETDA/TMC (red ●) and with DETDA/TMC (blue ■); Reference data (black ▲) are shown in Table S5. The red line is the 2015 polymer upper bound. (b) Transport properties of CMS membranes using mixed gases: H_2_/CH_4_, one day after pyrolysis. (c, d) Transport properties of CMS membranes comparison before and after aging using mixed gases: without DETDA/TMC (c) and with DETDA/TMC (d). [T = 35°C, P_up_ = 100 psi, P_down_ = 7 Torr, mixture composition (10% H_2_/90% CH_4_ (mol/mol)), number of membranes test (*n* = 3)]

Of course, mixed‐gas separation performance for H_2_/CH_4_ (10:90, molar ratio) is most relevant and is shown in Figure 3b for samples one day after pyrolysis. The CMS membrane modified with dual DETDA/TMC and VTMS treatments exhibited higher permeance (*P*/l_*H2*_ = 19.9 *GPU*, shown in Table S4) compared with the counterpart treated with only VTMS treatment (*P*/l_*H2*_ = 16.4 *GPU*, also in Table S4). The thinner dense separation layer from dual DETDA/TMC and VTMS treatment in Figure S2b gives higher 19.9 GPU H_2_ permeance vs. 16.4 GPU for only VTMS treatment. The dual treatment case shows a minor H_2_/CH_4_ mixed gas selectivity change from 2136.1 to 2062.9, giving a downstream hydrogen purity of 99.56 mol%, which is a desirable level. Moreover, as shown in Figure 3c,d, and Table S6, for the dual‐treated case, the 24‐day aged sample gave an even more appealing selectivity of 2,639.8 and 15.6 GPU H_2_ permeance, surpassing the performance of the VTMS‐only treated sample in Table S6.

### 2.2 He/CH_4_ Separation

Relative to the previously reported CMS (He permeance ∼ 3 GPU) [4], the VTMS‐treated CMS achieves 18.6 GPU. Despite this increased permeance, VTMS introduces minor imperfections, reducing He/CH_4_ selectivity from 16 700 [4] to 6198 (Table S7) based on pure gas feeds. Additional DETDA/TMC cross‐linking further exacerbates these imperfections, lowering He/CH_4_ pure gas selectivity to 2,611 (Table S7). Although both CMS membranes (one treated with only VTMS and the other treated with DETDA/TMC and VTMS) fall below the perfluoropolymer upper bound (Figure 4a) [27], their ability to withstand high pressures makes them promising for high‐pressure helium recovery applications. Of course, mixed gas performance is the bottom‐line consideration.

**FIGURE 4:**
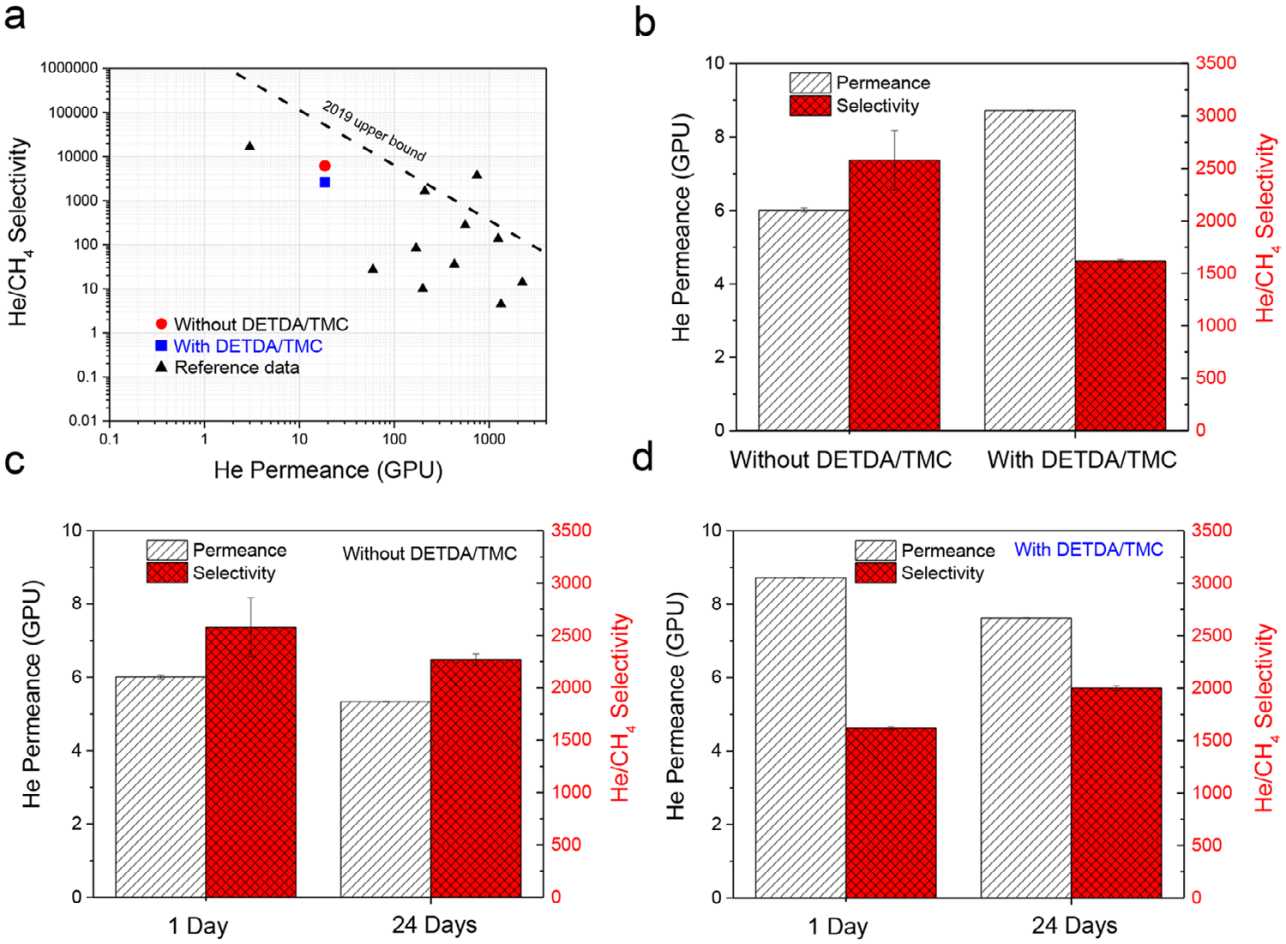
Pure gas separation performance comparison for VTMS‐treated precursor‐derived CMS membranes: (a) without DETDA/TMC (red ●) and with DETDA/TMC (blue ■); Reference data (black ▲) are shown in Table S13. Transport properties of CMS membranes using mixed gases: (b) He/CH_4_; Transport properties of CMS membranes comparison before and after aging using mixed gases: (c) without DETDA/TMC, and (d) with DETDA/TMC.

We evaluated membrane performance using a mixed feed of 5 mol% He and 95 mol% CH_4_. As shown in Figure 4b, the DETDA/TMC modified CMS exhibited a higher He permeance (*P*/l *_He_* = 8.7 *GPU*; Table S11) than the unmodified counterpart (*P*/l *_He_* = 6.0 *GPU*; Table S11). This gain is consistent with the thinner dense separation layer, which facilitates faster permeation of He through the matrix. Correspondingly, the He/CH_4_ mixed‐gas selectivity decreased from 2578.9 to 1617.8 (Table S11). Despite this trade‐off, the He product purity declined only slightly, from 99.3% to 98.8%, and remains at a highly desirable level for practical use.

### 2.3 Membrane Stability Test

Membrane stability was assessed by vacuum aging at 1 torr and 35°C for 24 days (Tables S8 and S9). Pure gas permeation declined for both membranes. The VTMS‐only CMS showed a 29.0% decrease in pure He gas permeance, from 18.6 to 13.2 GPU. On the other hand, the DETDA/TMC+VTMS CMS exhibited a much smaller decline of 5.9% (from 18.6 to 17.5 GPU). CH_4_ permeance also decreased in both cases, but more strongly for the DETDA/TMC+VTMS CMS (38.0%, 0.0071 to 0.0044 GPU) than for the VTMS‐only CMS (13.3%, 0.0030 to 0.0026 GPU). These trends are consistent with aging‐induced densification of the CMS, whereby initially imperfectly packed, disordered carbon strands compact, shrinking inter‐strand slits and reducing ultramicropore spacing [28]. In the VTMS‐only CMS, the CH_4_‐accessible pore volume is already limited. Further compaction therefore has a modest effect on CH_4_ but still reduces the He‐accessible ultramicropores, leading to a larger fractional drop in He permeance. By contrast, DETDA/TMC cross‐linking introduces additional imperfections within the selective Langmuir domains, likely by perturbing carbon plate settling during carbonization. Fortunately, controlled aging tightens these imperfections into narrower pathways that more effectively exclude CH_4_ while remaining permeable to He. Consequently, the DETDA/TMC+VTMS CMS experiences a pronounced reduction in CH_4_ permeance but only a minor loss in He permeance, indicating improved stability of He transport with aging. Mixed gas performance before and after aging is shown in Figure 4c,d. For the VTMS‐only CMS, He permeance decreased from 6.0 to 5.3 GPU after 24 days (Tables S11 and S12), accompanied by a reduction in He/CH_4_ selectivity from 2578.9 to 2271.5, mirroring the pure‐gas trend. Consequently, the He product purity declined slightly from 99.26% to 99.17%. In contrast, the DETDA/TMC+VTMS CMS exhibited an increase in He/CH_4_ selectivity from 1617.8 to 2000.8 despite a decrease in He permeance from 8.7 to 7.6 GPU. This tightening translated to a small improvement in He product purity, from 98.84% to 98.96%. These results indicate that aging slightly reduces He/CH_4_ separation performance of VTMS‐only CMS membranes, while it selectively enhances CH_4_ rejection in the DETDA/TMC‐cross‐linked CMS (Figure 4c,d).

### 2.4 Membrane Evolution During Aging

To elucidate membrane evolution during aging, we employed probe gases of varying kinetic diameters to interrogate pore structure and correlate it with performance. The gas transport behaviors of CMS membranes were probed through single‐gas permeation experiments. As shown in Figure 5a,b, the gas permeance exhibits a distinct cutoff between smaller and larger penetrant molecules in both VTMS‐only and DETDA/TMC‐modified CMS membranes. This observation suggests that permeation is primarily governed by the kinetic diameter of the penetrant gases, consistent with a molecular sieving transport mechanism.

**FIGURE 5:**
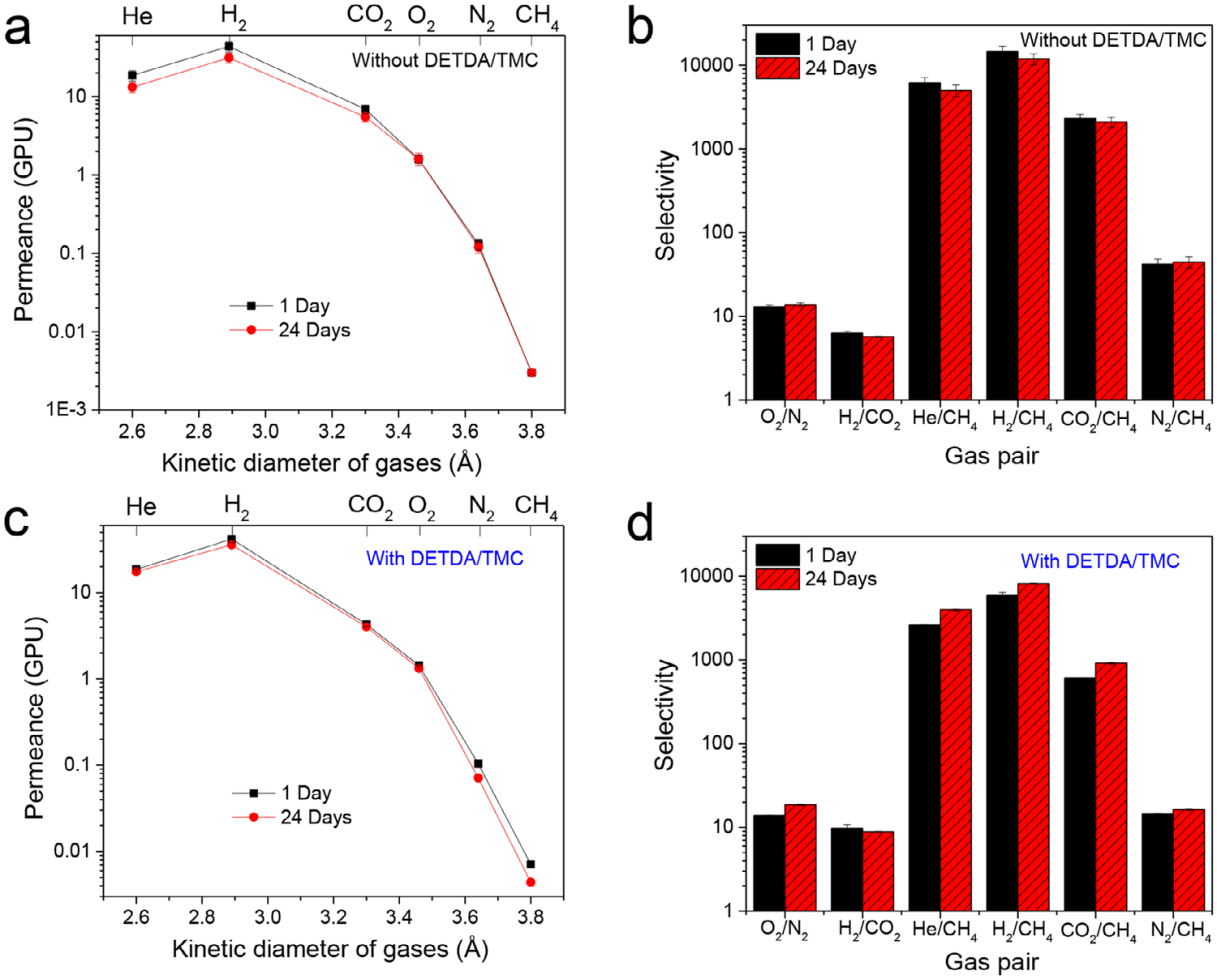
Transport properties of CMS membranes for pure gases before and after aging: (a) Pure gas permeance of different gases with VTMS‐only, (b) selectivities of different gas pairs with VTMS‐only, (c) Pure gas permeance of different gases with DETDA/TMC and VTMS, (d) selectivities of different gas pairs with DETDA/TMC and VTMS.

According to Table S8, the permeance of small gases such as H_2_ and He remains largely unaffected by the modification. In contrast, larger gases including CO_2_, O_2_, and N_2_ display reduced permeance after DETDA/TMC treatment, indicating a tightening of pore structures. This effect can be attributed to structural rearrangements, presumably due to shorter carbon strands from stress‐induced chain scission of the cross‐linked network precursor, which can be packed closer and reduce the accessibility for the penetrants such as H_2_, He, CO_2_, O_2_, and N_2_ molecules. At the same time, partial retention of cross‐linked branches during carbonization may slightly disrupt carbon strand packing, creating imperfections within the selective Langmuir domains [29] large enough for CH_4_ to pass through. Although their population is relatively small, these pores play a key role in increasing CH_4_ permeance, offering an additional transport pathway distinct from standard size‐sieving (Figure 5c,d).

Pure gas selectivities of different gas pairs are illustrated in Figure 5b,d. For all gas pairs involving CH_4_, a noticeable decrease in selectivity was observed for the DETDA/TMC treated samples, attributed to the substantial increase in CH_4_ permeance. This trend further reflects the influence of slit bypass pores facilitating CH_4_ transport. In contrast, selectivities for the medium‐sized pair O_2_/N_2_ increased, indicating enhanced molecular sieving. This improvement is ascribed to the tighter packing of short carbon strands formed after the decomposition of the cross‐linked polymer network during DETDA/TMC modification, which constrains the diffusion of larger penetrants more effectively.

All membranes showed some permeance decline after aging, with the VTMS‐only CMS exhibiting larger drops for small molecules (H_2_: 28.5%, He: 29.0%) than the DETDA/TMC‑modified membrane (H_2_: 14.1%, He: 5.9%), indicating a significant loss of some ultramicropores after aging. In contrast, DETDA/TMC‐modified CMS membranes displayed greater decline for larger molecules (N_2_:31.7%, CH_4_: 38.0%, as shown in Figure 5c). We hypothetically attribute this trend to the tightening of imperfections within the selective Langmuir domains during aging, thereby altering the pore size distribution compared with non‑cross‐linked precursor‐derived CMS membranes. After aging, the idealized micropore structure with imperfectly packed and disordered strands became more compact, with inter‐strand slits shrinking and the spacing being reduced [28]. This presumably led to a decrease in the number of imperfections within the selective layer for CH_4_. For the DETDA/TMC‐modified CMS membranes, the pure gas selectivity of He/CH_4_ increased significantly from 2610.9 to 3974.1 after aging. Consequently, high‐purity products (98.96% He) were obtained using mixed gas feeds. Notably, the DETDA/TMC‐modified CMS membranes maintained relatively high mixed gas permeances after aging (7.6 GPU He). Note that high surface to volume small diameter fibers can produce a very appealing tool for He recovery.

For H_2_/CH_4_ and He/CH_4_ separations, the exceptionally high selectivity demonstrated in this work is important for producing ultrahigh‐purity gas streams, which may be difficult to achieve using membranes with moderate selectivity. These results provide proof of concept for high‐purity gas production using CMS membranes in a single‐stage process. However, multistage process design and techno‐economic analysis are required to quantify their potential benefits.

The results discussed above demonstrate that DETDA/TMC treatment is a vital precursor‐engineering step for enhancing the performance and stability of the resultant CMS membrane. Although VTMS‐only modification provides a useful baseline and can exhibit strong initial single‐gas separation performance, DETDA/TMC cross‐linking produces a chemically transformed precursor that promotes the formation of a distinct carbon structure during pyrolysis and subsequent aging. This modification improves mixed‐gas H_2_/CH_4_ separation and enables favorable aging‐induced increases in selectivity, particularly for He/CH_4_ separation. Therefore, the role of DETDA/TMC extends beyond initial performance enhancement: it is essential for developing a more stable CMS structure with improved separation performance under practically relevant, aged mixed‐gas conditions.

## 3 Conclusion

In summary, we present a method to fabricate a chemically modified polymer precursor for hollow fiber membranes by treating economically conventional precursor fibers, thereby overcoming structural collapse and permeability limitations during high‐temperature carbonization. The DETDA/TMC strategy, applied to already defect‐free asymmetric hollow fibers, enables broad structural tunability and precursor innovation. Pure‐gas permeation measurements using probe molecules with different kinetic diameters were used to indirectly assess the active pores in the hollow‐fiber CMS selective layer. The DETDA/TMC treatment suppresses transition‐layer collapse during pyrolysis, producing a thinner, less densified carbon layer with reduced mass‐transfer resistance and enhanced transport. Our focus here showed clear mixed‐gas H_2_/CH_4_ and He/CH_4_ separations for realistic process conditions directly relevant to hydrogen purification and natural gas upgrading. Clearly, this strategy can likely be extended to diverse families of CMS polyimide precursors, broadening its tunable applicability across many other important gas pairs as well. The synergy between sol–gel stabilization and tailored DETDA/TMC modification provides a versatile platform for tuning stable CMS microstructures to enable point‐of‐use hydrogen purification in pipeline‐blending and other diverse cases. Clearly, using different types and amounts of diamine and acid chloride is possible and opens the way even beyond the new generation of high‐efficiency CMS membranes H_2_/CH_4_ and He/CH_4_ discussed here. Indeed, we envision the platform discussed here will have major positive impacts across the full spectrum of challenging gas pair separations.

## Author Contributions

Y. C. and W. J. K. conceived the idea and designed the experiments. Y. C. spun and modified the precursor fiber membranes, prepared CMS fiber membranes, performed the measurements of CMS fiber performance, characterized the precursor and CMS membranes and wrote the first draft of manuscript. Z. L., Z. S., J. K. and S. S. assisted in permeation measurement and data analyses. R. P. Lively reviewed and edited the manuscript. W. J. K. guided the whole work and finalized the manuscript. All authors contributed to writing and revising the manuscript.

## Conflicts of Interest

The authors declare no conflicts of interest.

## Supporting information




**Supporting File**: advs78223‐sup‐0001‐SuppMat.docx.

## Data Availability

The data that supports the findings of this study are available from the corresponding author upon reasonable request.
