# Supplementary material for: Transforming Gas Separations with Scalable Carbon Molecular Sieve Membranes Enabled by Chemically Tuned Hollow‐Fiber Precursors
Source: Adv Sci (Weinh). 2026 Oct 9:e78223. Online ahead of print. doi: 10.1002/advs.78223 (PMC13652583; doi:10.1002/advs.78223)
Supplement: Supplementary file 1 [file ADVS-9999-e78223-s001.docx]

Supporting Information

**Transforming Gas Separations with Scalable Carbon Molecular Sieve Membranes Enabled by Chemically Tuned Hollow-Fiber Precursors**

Yuhe Cao ^[a,b]^, Zhongyun Liu ^[c]^, Zahra Shomali ^[a]^, Jinyoung Kim ^[a]^, Steven Schlosser ^[a]^, Ryan P. Lively ^[a]^, William J. Koros*^[a]^

[a]	Y. Cao, Z. Liu, Z. Shomali, J. Kim, S. Schlosser, R. P. Lively, W. J. Koros

School of Chemical & Biomolecular Engineering,

Georgia Institute of Technology,

311 Ferst Drive, Atlanta, GA 30332, USA

E-mail: wjk@chbe.gatech.edu

[b]	Y. Cao

Department of Civil, Environmental and Construction Engineering

The University of Texas at El Paso, El Paso, TX 79968, USA

[c]	Z. Liu

Key Lab of Functional Polymers for Sustainability of Jiangsu

School of Energy and Environment

Southeast University, Nanjing, Jiangsu, P.R. China

**Experimental Procedures**

**Materials**

Matrimid® 5218-1 (BTDA-DAPI), a commercially available polyimide, was obtained from Huntsman International LLC. Prior to use, the polymer was dried overnight under vacuum at 110 °C. Vinyltrimethoxysilane (VTMS, 97% purity), hexane, 1-methyl-2-pyrrolidinone (NMP), trimesoyl chloride (TMC), ethanol, tetrahydrofuran (THF), and acetone were all sourced from Sigma-Aldrich (St. Louis, MO) in sure-seal bottles. Diethyltoluenediamine (DETDA) was purchased from Albemarle Corporation (Charlotte, NC). For polymer fiber solvent exchange, methanol (20 L) and hexane (20 L) were acquired from BDH Chemicals Co. Pure gases including He, H_2_, CO_2_, O_2_, N_2_, and CH_4_ (Research Grade) were obtained from Airgas (Radnor Township, PA), along with premixed gases of 10:90 (mole ratio) H_2_:CH_4_ and 5:95 (mole ratio) He:CH_4_ (±1% blend accuracy). All fittings for module fabrication were sourced from Swagelok® Georgia.

**Formation of precursor membranes**

Asymmetric hollow fiber membranes were prepared using a dry-jet/wet-quench spinning technique, with the experimental setup illustrated in Supplementary Figure S4. The spinning process utilized a Matrimid^®^ 5218-1 dope solution along with a bore fluid, the compositions of which are detailed in Supplementary Tables S14 and S15, while Table S15 also lists the specific spinning parameters. Following fiber formation, the membranes were immersed in water baths for three days to ensure complete removal of residual solvent. Subsequently, the fibers underwent solvent exchange in containers, first in three separate 30-minute methanol baths, followed by three separate 30-minute hexane baths. Finally, the fibers were dried under vacuum at 75 °C for 3 hours.

**DETDA/TMC modification of precursor fibers**

The precursor fiber membranes were first immersed in a 2% DETDA solution in hexane for 12 hours. After draining the DETDA solution, the fibers were rinsed in hexane for 30 minutes, repeated three times, to remove any residual DETDA. Next, the fibers were soaked in a 2% TMC solution in hexane for 1 hour. After draining the TMC solution, the fibers were again rinsed in hexane for 30 minutes. Finally, the fibers were treated with a 10% VTMS solution in hexane, followed by the VTMS treatment steps described below.

**VTMS treatment of fiber membranes and carbon molecular sieve membrane formation**

The VTMS treatment and pyrolysis procedures followed the same protocols as described in our previous work.[1] Initially, the fiber membranes were immersed in a 10 wt.% VTMS/hexane solution for 24 h and subsequently exposed to moisture at 25 °C. The VTMS-saturated fibers were then left under ambient conditions for approximately 24 h to promote the formation of crosslinked silica. Prior to pyrolysis, the silane-treated fibers were heated under vacuum at 150 °C for 12 h to remove any residual VTMS and moisture. Carbon molecular sieve (CMS) hollow fiber membranes were then produced by pyrolyzing the precursor fibers using a setup identical to that reported previously,[2] shown in Supplementary Figure S5. The heating protocol comprises: 1) 50 °C to 250 °C (13.3 °C /min); 2) 250 °C to 885 (3.85 °C /min); 3) 885 °C to 900 °C (0.25 °C /min); 4) Thermal soak at 900 °C for 120 min. After completion of the heating protocol, the furnace was allowed to cool naturally to room temperature while maintaining a flow of UHP argon. Once the CMS hollow fiber membranes were formed, membrane modules were assembled using Scotch-Weld DP-100 epoxy resin (3M Corporation), following procedures reported in earlier studies.[3]

**Characterization**

Thermogravimetric analysis (TGA) combined with evolved gas analysis by Fourier transform infrared (FTIR) spectroscopy was carried out using a TA Instruments Q50 thermogravimetric analyzer interfaced with a Nicolet iS50 infrared spectrometer. The thermal program employed was identical to that applied during the pyrolysis procedures. Also, ATR–FTIR spectra (Thermo Scientific, iS50) of the precursor fibers, both before and after DETDA/TMC treatment, were recorded at a resolution of 2 cm⁻¹ with 64 scans per measurement. The XPS spectra of membranes were recorded on a Thermo K-Alpha XPS with an X-ray beam size of 100 μm. Zeiss Ultra60 Fe-SEM was used to characterize the morphologies of polymer precursor hollow fibers and CMS hollow fibers.

**Pure-gas and mixed-gas permeation**

Pure gas permeation experiments were performed at 35 °C using a variable-pressure, constant-volume apparatus, as outlined in previous studies.[4] CMS hollow fiber membranes were evaluated for single-gas permeation with He, H_2_, CO_2_, O_2_, N_2_, and CH_4_ at a feed pressure of 100 psia, with vacuum maintained on the permeate side. Mixed-gas tests were conducted under identical conditions for 10/90 mol% H_2_/CH_4_ mixtures and 5/95 mol% He/CH_4_ mixtures, following the methodology described in our previous work.[4] A crossflow configuration was implemented on the feed side, while the downstream remained under vacuum to avoid concentration polarization. Feed flow was precisely controlled via a stainless-steel metering valve, and the stage cut (permeate/feed flow ratio) was limited to ≤1%. Prior to data acquisition, membranes were equilibrated for at least 8 hours to ensure steady-state conditions. Once stabilized, the downstream vacuum valve was sealed, and pressure changes in the permeate side were monitored over time. Gas composition in the permeate was analyzed using a Bruker- 450-GC gas chromatograph.

For each pure and mixed gas pair, triplicate CMS samples were tested. The pure gas permeance (P/l) of the CMS membrane, expressed in GPU (1 GPU = 3.35 × 10⁻¹⁰ mol·m⁻²·s⁻¹·Pa⁻¹), was calculated using Equation (1):

$P/l=6.949\times{10}^{4}\times\frac{\left( \frac{\Delta p}{\Delta t} \right)\times V_{down}}{A\times T\times\Delta f}$	(1)

where Δp/Δt is the slope of downstream pressure versus time (Torr·s⁻¹); *l* is the membrane selective layer thickness (µm); *V*_down_ is the downstream system volume (cm³); *A* is the membrane area (cm²); *T* is the testing temperature (K); and Δ*f* is the fugacity difference between the upstream and downstream sides (psi), determined using the Peng–Robinson equation of state in combination with the SUPERTRAPP program developed by NIST.

The permeance (*Pi/l*) of a membrane for mixed gases was calculated using Equation (2):

$P_{i}/l=6.949\times{10}^{4}\times\frac{\left( \frac{\Delta p}{\Delta t} \right)\times y_{i}{\times V}_{down}}{A\times T\times(x_{i}f_{upstream}-y_{i}f_{downstream})}$	(2)

where *yᵢ* is the molar fraction of component *i* in the permeate, determined using a Bruker-450 gas chromatograph. In Equation (2), *xᵢ* represents the molar fraction of component *i* in the feed (upstream). *f*_upstream_ and *f*_downstream_ denote the fugacities of the feed (upstream) and permeate (downstream), respectively, expressed in psi. These fugacities were calculated using the Peng–Robinson equation of state in conjunction with the SUPERTRAPP program developed by NIST.

The selectivity (*α_i,j_*) is calculated by the ratio of the permeance of component *i* to the permeance of component *j* (equation (3)):

$\alpha_{i,j}=\frac{P_{i}/l}{P_{j/l}}$	(3)

**Figure S1.** SEM images of CMS hollow fiber membrane pyrolyzed without VTMS stabilization treatment: (a) fiber overview, (b) fiber wall, and (c) fiber top surface. As shown by (b), the CMS hollow fiber wall was entirely dense; hence the separation layer thickness equals the fiber wall thickness (~32 μm).[5]

**Figure S2.** Comparison of the top separation layers of the CMS membranes. (a) without DETDA+TMC; (b) with DETDA+TMC modification to reduce selective layer thickness.

**Figure S3.** SEM images of 900 ℃ CMS membranes from 10% VTMS treated monolithic precursor fibers without DETDA/TMC. (a) cross section; (b) fiber wall; (c) top separation layer.

**Figure S4.** Dry-jet/wet-quench process for producing precursor hollow fiber membranes.[1]

**Figure S5.** Schematic representation of the pyrolysis system.[1]

**Table S1.** Elemental compositions (at%) of fiber membranes.

|  | Without DETDA/TMC | With DETDA/TMC |
| --- | --- | --- |
| C1s | 79.2 | 71.5 |
| O1s | 14.9 | 18.5 |
| N1s | 5.9 | 8.0 |
| Cl | ---- | 2.0 |
| O/C | 0.19 | 0.26 |
| N/C | 0.07 | 0.11 |

**Table S2**. The curve fitting results of C1s of fiber membranes.

|  | Relative content (%) | |
| --- | --- | --- |
|  | Without DETDA/TMC | With DETDA/TMC |
| C-C (284.8 eV) | 34.2 | 31.4 |
| C-H (285.5 eV) | 39.1 | 28.3 |
| C-N (286.4 eV) | 14.2 | 26.0 |
| C=O (288.7 eV) | 9.3 | 11.7 |
| π-π* shake-up satellite peak (291.3 eV)) | 3.2 | 2.6 |

**Table S3.** Pure gas permeation performance of the CMS membranes (Tested at 35 ℃ with 100 psia feed pressure).

| Membranes | H_2_  (GPU) | α_H2/CH4_ |
| --- | --- | --- |
| Without DETDA/TMC | 43.8±6.5 | 14,592.8±2174.6 |
| With DETDA/TMC | 41.9±3.9 | 5,891.7±548.3 |

**Table S4.** H_2_/CH_4_ unaged (1 day after pyrolysis) mixed gas permeation performance using different CMS membranes (Tested at 35 ℃ with 100 psia feed pressure).

| Membranes | P/_H2_(GPU) | α_H2/CH4_ | Downstream H_2_ purity  (%) |  |
| --- | --- | --- | --- | --- |
| Without DETDA/TMC | 16.4±0.1 | 2,136.1±566.0 | 99.55±0.13 |  |
| With DETDA/TMC | 19.9±0.1 | 2,062.9±235.4 | 99.56±0.05 |  |

**Table S5.** Separation performance of typical membranes for H_2_/CH_4_ separation.

| Membrane materials | | Test temperature (K) | H_2_ permeance (GPU) | H_2_/CH_4_ selectivity | Ref. |
| --- | --- | --- | --- | --- | --- |
| Zeolite | SSZ-13 | 298 | 13,432 | 82 | [6] |
|  | Sodalite SOD | 523 | 119 | 23 | [7] |
|  | Si-CHA | 298 | 4,300 | 68 | [8] |
| Graphene | Plasma-treated | 423.2 | 3,000 | 30 | [9] |
| MOF | 2D ZIF-95 | 373 | 2,866 | 64.3 | [10] |
|  | ZIF-8@GO | 523 | 432 | 145 | [11] |
| Polymer | PIM-1 | 308 | 350 | 21.9 | [12] |
|  | PIM-1/Matrimid^®^ | 308 | 395* | 116.2 | [13] |
|  | P33DT-ThC4 | 298 | 614* | 68.8 | [14] |
|  | PIM-BM/TB-300°C-5 h | 308 | 358* | 831.6 | [15] |
|  | b-oriented membrane | 393 | 342.2* | 117.1 | [16] |
|  | TPIM-1 | 308 | 1,105* | 152 | [17] |
|  | KAUST-PI-1 | 308 | 3,431* | 41 | [17] |
|  | PIM-TRIP-TB | 308 | 4,740* | 22 | [17] |
|  | PIM-EA-TB | 308 | 4,442* | 20 | [17] |
| CMS | CMS-900 | 303 | 0.29* | 1,859 | [18] |
|  | CMS | -- | 56.4 | 264.3 | [19] |
|  | CMS | 308 | 8^**^ | 40,350 | [5] |
|  | CMS | 308 | 239.6 | 662.6 | [20] |
|  | CMS | 403 | 148.2 | 5,706 | [21] |
|  | CMS-without DETDA/TMC | 308 | 43.8 | 14,593 | This work |
|  | CMS-with DETDA/TMC | 308 | 41.9 | 5,891.7 | This work |

Note: *assuming a 1-μm-thick selective layer; ^**^permeance is calculated by permeance=Permeability/membrane thickness for uniform comparison purposes

**Table S6.** H_2_/CH_4_ mixed gas permeation performance using different CMS membranes after 24 days (Tested at 35 ℃ with 100 psia feed pressure).

| Membranes | P/_H2_(GPU) | α_H2/CH4_ | Downstream H_2_ purity  (%) |  |
| --- | --- | --- | --- | --- |
| Without DETDA/TMC | 12.9±0.1 | 2,445.5±31.8 | 99.63±0.01 |  |
| With DETDA/TMC | 15.6±0.1 | 2,639.8±77.9 | 99.75±0.02 |  |

**Table S7.** Pure gas selectivities of different gas pairs (Tested at 35 ℃ with 100 psia feed pressure, day 1).

| Membranes | α_O2/N2_ | α_H2/CO2_ | α_He/CH4_ | α_H2/CH4_ | α_CO2/CH4_ | α_N2/CH4_ |
| --- | --- | --- | --- | --- | --- | --- |
| Without DETDA/TMC | 13.0±0.7 | 6.3±0.3 | 6197.6±941.9 | 14592.8±2174.6 | 2305.8±268.3 | 41.7±6.2 |
| With DETDA/TMC | 13.8±0.1 | 9.8±0.9 | 2610.9±15.3 | 5891.7±548.3 | 602.4±2.5 | 14.6±0.2 |

**Table S8.** Pure gas permeation performance of the CMS membranes (Tested at 35 ℃ with 100 psia feed pressure, day 1).

| Membranes | H_2_  (GPU) | He  (GPU) | CO_2_  (GPU) | O_2_  (GPU) | N_2_  (GPU) | CH_4_  (GPU) |
| --- | --- | --- | --- | --- | --- | --- |
| Without DETDA/TMC | 43.8±6.5 | 18.6±2.8 | 6.9±0.8 | 1.6±0.2 | 0.125±0.019 | 0.0030±0.0001 |
| With DETDA/TMC | 41.9±3.9 | 18.6±0.1 | 4.3±0.1 | 1.4±0.1 | 0.104±0.002 | 0.0071±0.0001 |

**Table S9.** Pure gas permeation performance of the CMS membranes after 24 days (Tested at 35 ℃ with 100 psia feed pressure).

| Membranes | H_2_  (GPU) | He  (GPU) | CO_2_  (GPU) | O_2_  (GPU) | N_2_  (GPU) | CH_4_  (GPU) |
| --- | --- | --- | --- | --- | --- | --- |
| Without DETDA/TMC | 31.3±4.8 | 13.2±2.1 | 5.5±0.8 | 1.6±0.3 | 0.120±0.020 | 0.0026±0.0001 |
| With DETDA/TMC | 36.0±0.7 | 17.5±0.6 | 4.0±0.1 | 1.3±0.1 | 0.071±0.002 | 0.0044±0.0001 |

**Table S10.** Pure gas selectivities of different gas pairs after 24 days (Tested at 35 ℃ with 100 psia feed pressure).

| Membranes | α_O2/N2_ | α_H2/CO2_ | α_He/CH4_ | α_H2/CH4_ | α_CO2/CH4_ | α_N2/CH4_ |
| --- | --- | --- | --- | --- | --- | --- |
| Without DETDA/TMC | 13.7±0.7 | 5.7±0.1 | 5009.7±801.8 | 11891.3±1808.4 | 2089.7±296.9 | 44.4±7.3 |
| With DETDA/TMC | 18.6±0.4 | 8.9±0.1 | 3974.1±132.4 | 8188.1±147.9 | 916.3±23.7 | 16.3±0.5 |

**Table S11.** He/CH_4_ mixed gas permeation performance using different CMS membranes (Tested at 35 ℃ with 100 psia feed pressure, day 1).

| Membranes | P/_He_(GPU) | α_He/CH4_ | Downstream He purity  (%) |
| --- | --- | --- | --- |
| Without DETDA/TMC | 6.0±0.1 | 2578.9±280.8 | 99.26±0.08 |
| With DETDA/TMC | 8.7±0.1 | 1617.8±14.1 | 98.84±0.01 |

**Table S12.** He/CH_4_ mixed gas permeation performance using different CMS membranes after 24 days (Tested at 35 ℃ with 100 psia feed pressure).

| Membranes | P/_He_(GPU) | α_He/CH4_ | Downstream He purity (%) |
| --- | --- | --- | --- |
| Without DETDA/TMC | 5.3±0.1 | 2271.5±55.2 | 99.17±0.02 |
| With DETDA/TMC | 7.6±0.1 | 2000.8±21.5 | 98.96±0.05 |

**Table S13.** Comparison of the He/CH_4_ separation performance of this work with reported data.

| Membrane materials | Test temperature (K) | He permeance (GPU) | He/CH_4_ selectivity | Ref. |
| --- | --- | --- | --- | --- |
| ZSM-5 Zeolite | 298 | 2239 | 14 | [22] |
| 3.50% C_70_@ZIF-8 | 296 | 200 | 10 | [23] |
| SSZ-13 zeolite | 298 | 59.7 | 27.4 | [24] |
| Fluorinated PIM-1 | 308 | 745* | 3770 | [25] |
| PIM-1 | 308 | 1336* | 4.5 | [25] |
| Poly(PFMD) | 308 | 210* | 1650 | [26] |
| Poly(PFMMD) | 308 | 560* | 280 | [26] |
| Hyflon AD80 | 308 | 430* | 36 | [26] |
| Cytop | 308 | 170* | 84 | [26] |
| CMS | 308 | 3^**^ | 16700 | [5] |
| CMS | 308 | 1248.5 | 137.1 | [27] |
| CMS-without DETDA/TMC | 308 | 18.6 | 6198 | This work |
| CMS-with DETDA/TMC | 308 | 18.6 | 2611 | This work |

Note: *assuming a 1-μm-thick selective layer; **permeance is calculated by permeance=Permeability/membrane thickness

**Table S14.** Composition of the precursor spinning components.

| Sheath dope | Wt.% | Bore fluid | Wt.% |
| --- | --- | --- | --- |
| Matrimid^®^ | 26.2 | NMP | 96 |
| NMP | 53.0 | H_2_O | 4 |
| THF | 5.9 | -- | -- |
| EtOH | 14.9 | -- | -- |

**Table S15.** Spinning conditions of precursor membranes and O_2_/N_2_ separation performance. The temperatures of the pumps, inline, spinneret were maintained at 60 °C; coagulation bath temperature was 50 °C; gas feed pressure was 50 psi and the testing temperature was 35 °C.

| Dope Flow  (mL/hr) | Bore Flow  (mL/hr) | Air gap  (cm) | Takeup rate  (m/min) | N_2_ permeance  (GPU) | O_2_ permeance  (GPU) | α O_2_/N_2_ |
| --- | --- | --- | --- | --- | --- | --- |
| 120 | 120 | 5 | 14 | 3.5 | 22.3 | 6.4 |

| a | **b** |
| --- | --- |

**Figure S6.** (a) Permeation box leakage test: downstream pressure vs. time; (b) CH_4_ permeation test: downstream pressure vs. time.

**References**

[1] Y. Cao, K. Zhang, O. Sanyal, W.J. Koros, "Carbon Molecular Sieve Membrane Preparation by Economical Coating and Pyrolysis of Porous Polymer Hollow Fibers," Angew Chem Int Ed 58, no. 35 (2019): 12149-12153, https://doi.org/10.1002/anie.201906653.

[2] L. Xu, M. Rungta, W.J. Koros, "Matrimid® derived carbon molecular sieve hollow fiber membranes for ethylene/ethane separation," Journal of Membrane Science 380, no. 1-2 (2011): 138-147, https://doi.org/10.1016/j.memsci.2011.06.037.

[3] C.W. Jones, W.J. Koros, "Carbon molecular sieve gas separation membranes-I. Preparation and characterization based on polyimide precursors," Carbon 32, no. 8 (1994): 1419-1425, https://doi.org/10.1016/0008-6223(94)90135-X.

[4] Z. Liu, W. Qiu, W. Quan, Y. Liu, W.J. Koros, "Fine-tuned thermally cross-linkable 6FDA-based polyimide membranes for aggressive natural gas separation," Journal of Membrane Science 635, (2021): 119474, https://doi.org/10.1016/j.memsci.2021.119474.

[5] C. Zhang, W.J. Koros, "Ultraselective Carbon Molecular Sieve Membranes with Tailored Synergistic Sorption Selective Properties," Adv Mater 29, no. 33 (2017):1701631, https://doi.org/10.1002/adma.201701631.

[6] Y. Li, S. He, C. Shu, X. Li, B. Liu, R. Zhou, Z. Lai, "A facile approach to synthesize SSZ-13 membranes with ultrahigh N2 permeances for efficient N2/CH4 separations," J. Membr. Sci. 632, (2021): 119349, https://doi.org/10.1016/j.memsci.2021.119349.

[7] M. Dakhchoune, L.F. Villalobos, R. Semino, L. Liu, M. Rezaei, P. Schouwink, C.E. Avalos, P. Baade, V. Wood, Y. Han, M. Ceriotti, K.V. Agrawal, "Gas-sieving zeolitic membranes fabricated by condensation of precursor nanosheets," Nat. Mater. 20, (2021): 362-369, https://doi.org/10.1038/s41563-020-00822-2.

[8] T. Wu, C. Shu, S. Liu, B. Xu, S. Zhong, R. Zhou, "Separation Performance of Si-CHA Zeolite Membrane for a Binary H2/CH4 Mixture and Ternary and Quaternary Mixtures Containing Impurities," Energ. Fuel. 34, no. 9 (2020): 11650-11659, https://doi.org/10.1021/acs.energyfuels.0c01720.

[9] J. Zhao, G. He, S. Huang, L.F. Villalobos, M. Dakhchoune, H. Bassas, K.V. Agrawal, "Etching gas-sieving nanopores in single-layer graphene with an angstrom precision for high-performance gas mixture separation," Science Advances 5, no. 1 (2019): eaav1851, https://doi.org/10.1126/sciadv.aav1851.

[10] X. Ma, Z. Wan, Y. Li, X. He, J. Caro, A. Huang, "Anisotropic Gas Separation in Oriented ZIF-95 Membranes Prepared by Vapor-Assisted In-Plane Epitaxial Growth," Angew. Chem. Int. Ed. 59, no. 47 (2020): 20858-20862, https://doi.org/10.1002/anie.202008260.

[11] A. Huang, Q. Liu, N. Wang, Y. Zhu, J. Caro, "Bicontinuous zeolitic imidazolate framework ZIF-8@GO membrane with enhanced hydrogen selectivity," J. Am. Chem. Soc. 136, no. 42 (2014): 14686-14689, https://doi.org/10.1021/ja5083602.

[12] M.L. Jue, V. Breedveld, R.P. Lively, "Defect-free PIM-1 hollow fiber membranes," J. Membr. Sci. 530, (2017): 33-41, https://doi.org/10.1016/j.memsci.2017.02.012.

[13] W.F. Yong, F.Y. Li, T.-S. Chung, Y.W. Tong, "Highly permeable chemically modified PIM-1/Matrimid membranes for green hydrogen purification," J. Mater. Chem. A 1, no. 44 (2013): 13914, https://doi.org/10.1039/c3ta13308g.

[14] M. Zhang, X. Jing, S. Zhao, P. Shao, Y. Zhang, S. Yuan, Y. Li, C. Gu, X. Wang, Y. Ye, X. Feng, B. Wang, "Electropolymerization of Molecular-Sieving Polythiophene Membranes for H(2) Separation," Angew Chem Int 131, no. 26 (2019): 8768-8772, https://doi.org/10.1002/ange.201904385.

[15] X. Chen, Y. Fan, L. Wu, L. Zhang, D. Guan, C. Ma, N. Li, “Ultra-selective molecular-sieving gas separation membranes enabled by multi-covalent-crosslinking of microporous polymer blends”, Nat Commun 12, (2021) 6140, https://doi.org/10.1038/s41467-021-26379-5.

[16] S. Chen, M. Wahiduzzaman, T. Ji, Y. Liu, Y. Li, C. Wang, Y. Sun, G. He, G. Maurin, S. Wang, Y. Liu, “Oriented Titanium-MOF Membrane for Hydrogen Purification,” Angew Chem Int Ed 64, no. 1 (2025) e202413701, https://doi.org/10.1002/ange.202413701.

[17] R. Swaidan, B. Ghanem, I. Pinnau, “Fine-Tuned Intrinsically Ultramicroporous Polymers Redefine the Permeability/Selectivity Upper Bounds of Membrane-Based Air and Hydrogen Separations,” ACS Macro Lett 4, no. 9 (2015) 947-951, https://doi.org/10.1021/acsmacrolett.5b00512.

[18] R. Xu, L. He, L. Li, M. Hou, Y. Wang, B. Zhang, C. Liang, T. Wang, “Ultraselective carbon molecular sieve membrane for hydrogen purification,” J. Energy Chem. 50, (2020) 16-24, https://doi.org/10.1016/j.jechem.2020.03.008.

[19] J. Hou, H. Zhang, Y. Hu, X. Li, X. Chen, S. Kim, Y. Wang, G.P. Simon, H. Wang, “Carbon Nanotube Networks as Nanoscaffolds for Fabricating Ultrathin Carbon Molecular Sieve Membranes,” ACS Appl. Mater. Interfaces 10, no. 23 (2018) 20182-20188, https://doi.org/10.1021/acsami.8b04481.

[20] Y. Cao, Z. Liu, W.J. Koros, “Above-T(g) Annealing Benefits in Nanoparticle-Stabilized Carbon Molecular Sieve Membrane Pyrolysis for Improved Gas Separation,” Angew Chem Int Ed 63, no. 8 (2024) e202317864, https://doi.org/10.1002/anie.202317864.

[21] L. Lei, F. Pan, A. Lindbrathen, X. Zhang, M. Hillestad, Y. Nie, L. Bai, X. He, M.D. Guiver, “Carbon hollow fiber membranes for a molecular sieve with precise-cutoff ultramicropores for superior hydrogen separation,” Nat Commun 12, (2021) 268, https://doi.org/10.1038/s41467-020-20628-9.

[22] Y. Deng, F. Qiu, J. Wu, J. Li, Z. Li, Q. Huang, G. Zhu, X. Zou, “Flexible ZSM-5 Zeolite Membrane for High-Performance Helium Separation,” Angew Chem Int Ed 64, no. 6 (2025) e202421285, https://doi.org/10.1002/anie.202421285.

[23] J. Han, H. Wu, H. Fan, L. Ding, G. Hai, J. Caro, H. Wang, “Tuning the Phase Composition of Metal-Organic Framework Membranes for Helium Separation through Incorporation of Fullerenes,” J Am Chem Soc 145, no. 27 (2023) 14793-14801, https://doi.org/10.1021/jacs.3c03362.

[24] J. Xie, T. Zhou, L. You, Y. Dai, Q. Zhou, C. Zhang, X. Wang, X. Gu, “Helium extraction from helium-poor natural gas using hollow fiber SSZ-13 zeolite membranes,” Journal of Membrane Science 735, (2025), https://doi.org/10.1016/j.memsci.2025.124524.

[25] X. Ma, K. Li, Z. Zhu, H. Dong, J. Lv, Y. Wang, I. Pinnau, J. Li, B. Chen, Y. Han, “High-performance polymer molecular sieve membranes prepared by direct fluorination for efficient helium enrichment,” Journal of Materials Chemistry A 9, no. 34 (2021) 18313-18322, https://doi.org/10.1039/d1ta04099e.

[26] M. Yavari, M. Fang, H. Nguyen, T.C. Merkel, H. Lin, Y. Okamoto, “Dioxolane-Based Perfluoropolymers with Superior Membrane Gas Separation Properties,” Macromolecules 51, no. 7 (2018) 2489-2497, https://doi.org/10.1021/acs.macromol.8b00273.

[27] C. Feng, H. Guo, M. Deng, J. Wei, Y. Ma, Z. Qin, X. Ma, J. Liu, L. Deng, W. Jiang, L. Yang, L. Yao, Z. Dai, Thin-Film-Composite Carbon Molecular Sieve Membranes for Efficient Helium and Hydrogen Separation, Industrial & Engineering Chemistry Research 63, no. 1 (2023) 594-606, https://doi.org/10.1021/acs.iecr.3c03687.
